# Catching the Mardi Gras fever: Quantifying the impact of mass gathering tourism on local bacterial prevalence and community diversity in municipal wastewater

**DOI:** 10.21203/rs.3.rs-8681977/v1

**Published:** 2026-02-02

**Authors:** Joe Berta, Lorri Rowe, Bob Garry

**Affiliations:** Tulane University; Tulane University; Tulane University

## Abstract

We employed 16S metagenomic analysis to measure the impact of Mardi Gras tourism on the bacterial ecology found in New Orleans’ municipal wastewater. Throughout the peak of the 2023 Carnivale season, species turnover was significantly higher in New Orleans than it was in our control site. Alpha diversity metrics peaked 2-to-3 weeks after Mardi Gras Day, increasing between 65% and 1967% over Carnivale. We also found that human pathogens and microbiota had significantly stronger, more positive correlations with the rise in Mardi Gras tourism than did environmental control species. These changes in wastewater abundance for two species – *S. enterica* and *E. coli* – mirrored the concurrent clinical isolate data from the same region for *Salmonella spp*. and STEC. We also found that multiple alpha and beta diversity measures correlated strongly with increases in tourism during the peak of Carnivale season.

## Introduction

Despite over a century of application in public health, bacterial wastewater surveillance is often overlooked as a tool for tracking the prevalence of bacteria.^1–3^ Monitoring of bacterial pathogens and their outbreaks still occurs in wastewater epidemiology, but studies are sporadic compared to that of viruses, with most bacterial surveillance happening at the clinical level or via a handful of indicator species.^4–6^ Generally, these epidemiological wastewater studies are conducted using targeted, quantitative molecular methods, such as qPCR, or culture.^4,5,7,8^ Meanwhile, untargeted sequencing and 16S metabarcoding have been employed only in a limited capacity for such purposes.

While many are familiar with New Orleans’s Mardi Gras celebration, Mardi Gras Day is only the final climax of the two-month Carnivale season. There are around forty official parades throughout Carnivale season, culminating in almost daily parades for the final two weeks.^9^ Studies have estimated that Orleans Parish – local population of 364 thousand – receives 1–1.4 million tourists during Carnivale.^10–12^ Tourism is mostly centered on the final weeks of the Carnivale season, with New Orleans hotels maintaining an average 74.2% capacity rate (18,763 rooms) throughout the closing two weeks of the 2023 season, spiking to 84.5% (21,331 rooms) and 93.5% (23,656 rooms) capacity the last two weekends.^13^ With the majority of parade-goers coming from outside the city of New Orleans (56.2%), this temporary doubling of the Orleans Parish population makes Mardi Gras as deserving of consideration as more well-studied mass gatherings, e.g. the Kumbh Mela, the Hajj, and various music festivals and sporting events.

We test the ability of 16S metabarcoding to track individual pathogens, antibiotics resistance genes (ARGs), and human microbiota. Wastewater concentrations of *E. coli*, *K. pneumoniae*, *P. aeruginosa*, and enterococci, have all been strongly correlated with the concentration of ARGs in a wastewater treatment plant (WWTP), making 16S a promising option for ARG surveillance to indicate when targeted, follow-up methods are warranted.^14,15^

Studies have found a robust correlation between the concentrations of several bacterial pathogens in wastewater and their incidence in clinical isolates: *S. enterica*, *C. trachomatis, T. pallidum*, and *E. coli*, etc.^4,16–22^ Tying bacterial sewage concentrations to clinical case rates is necessary as, unlike viruses, many disease-causing bacteria are facultative or opportunistic pathogens, meaning they can often reproduce without infecting a human host.^14,23^ We use the CDC’s BEAM dashboard to verify correspondence between wastewater abundance and clinical incidence for two species: *E. coli* and *S. enterica*.^24^

Finally, we propose and test a protocol for the study of mass gatherings using wastewater surveillance: employing matched sampling of a control WWTP to negate the variation weather induced on the sewage bacterial communities. Since the study of mass gathering medicine has produced several effective public health measures to ameliorate the risk of infectious disease during mass gatherings, tracking the extent and duration of their impact on circulating diseases yields actionable data that could aid in lowering the disease burden at the sites of our most beloved festival, religious, and sporting traditions.^25–29^

## Online Materials and Methods

### Sample Collection

“North Shore” samples were collected from the Mandeville Public Works WWTP in Mandeville, Louisiana, which has a catchment population of around thirteen thousand. “NOLA” or “New Orleans” samples were collected from Veolia East Bank WWTP located in New Orleans, Louisiana. The New Orleans East Bank has an approximate catchment population of 330 thousand. From January 12th to March 16th, 2023, one-liter, twenty-four-hour composite samples were collected from New Orleans and the North Shore each Thursday by autosamplers from influent wastewater.

### Wastewater Sample Processing

Samples were frozen at −80°C until they could be sequenced. Samples were first thawed and homogenized. Large solids were removed via tabletop centrifuge at 500 × g for five minutes. Samples were concentrated via high-speed centrifugation. The prokaryotic fraction was selectively pelleted from the resulting supernatant at an RCF_AVE_ of 12,000 × g for fifteen minutes. Samples were then stored at 4°C overnight. Prokaryotic DNA extraction was conducted according to the Zymo Quick-DNA/RNA Miniprep Plus Kit and pre-sequencing quality control was confirmed via Nanodrop OneC.

### Library Prep and 16S Sequencing

Library preparation for 16S rRNA sequencing was conducted using the Illumina MiSeq “16S Metagenomic Sequencing Library Preparation” protocol.^30^ Samples were sequenced on an Illumina MiSeq using a V2 Reagent Kit generating circa 118k reads per sample.

### Read Process and Analysis

Initial sequencing read quality control was conducted using FastQC. Bases with a Phred below Q20, truncated reads and adapter sequences were trimmed using Cutadapt version 4.8.^31,32^ 16S Metagenomic analysis was conducted using Kraken 2.^33^ For Kraken 2, demultiplexed, trimmed reads were input as FASTQ files into Kraken 2 version 2.1.3. The Standard 8GB Kraken reference database was used with default parameters except for the confidence threshold, which was raised to 0.01. Read mapping selectivity was optimized by removing from consideration all detected taxa that had fewer than 0.004% of the total number of reads mapped to them. Resultant taxonomic reports were then fed into Bracken v. 2.9.^34^ Species-level taxonomic reports were generated using 250 bp-length reads along with default settings.

### Alpha and Beta Diversity Calculations

All alpha diversity measures were calculated using the “Diversity” package within Scikit-Bio.^35^ Samples’ species richness was calculated using a simple Observed Taxonomic Unit (OTU) count. Evenness was calculated using Pielou’s Evenness and Simpsons Evenness Index, which is calculated by dividing the Gini-Simpson index by the total number of taxa.^36–38^ Overall diversity was estimated using Shannon Entropy – calculated using the Shannon-Weiner Index – and the Gini-Simpson index subtracted from 1.^37–39^ The Bray-Curtis dissimilarity formula was used to score the beta diversity between timepoints.^40^

### Significance Testing

Student’s t-test, one-way ANOVAs and Pearson R calculations and significance testing was conducted using SciPi’s “stats” library.^41^ Linear and multiple regressions were conducted using the OLS() function in the statsmodels.api library.^42^

### Historical Weather and Clinical Isolate Data

All weather data was found via the National Centers for Environmental Information’s online Past Weather database and search function, found at https://www.ncei.noaa.gov/access/past-weather/. Clinical isolate data was collected using the CDC’s BEAM dashboard found at https://www.cdc.gov/ncezid/dfwed/BEAM-dashboard.html.

## Results

### Controlling conflicting variation from weather

Pearson correlation analysis showed that New Orleans and the North Shore both had a strong, positive correlation between their weekly temperatures (High: R = 0.845; Low: R = 0.969, Average: R = 0.952) and weekly precipitation (Pearson, R = 0.991) during the period of study. (**see Supp.** Figure 1, **Supp.** Table 1) Paired t-tests determined that the sample means from each site for each component of weather were statistically similar. (High Temp: p = 2.08*10^− 3^; Low Temp: p = 3.88*10^− 6^, Average Temp: p = 2.12*10^− 5^, Precipitation: 3.21*10^− 8^)

### Dominant taxa turnover

**Figure 1:**
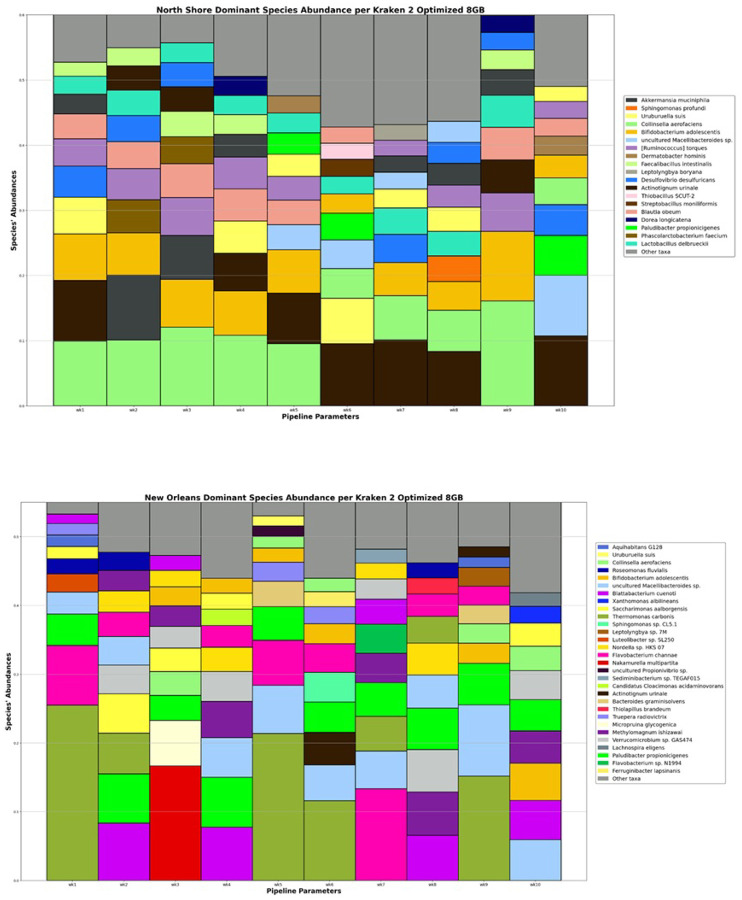
Change in dominant taxa throughout Carnivale. Relative abundance of dominant taxa per week according to each pipeline bars representing the ten most dominant bacterial species at each timepoint throughout for (top) New Orleans and (bottom) the North Shore. Combined abundance of all other taxa represented in grey.

We plotted the abundance of the ten most dominant taxa for each time point at both sampling locations. (**see** Fig. 1, **Supp. Table 2**) For weekly Bray-Curtis species turnover, New Orleans averaged 0.559, while the North Shore averaged 0.316. These weekly species turnover differences were found to be significantly different. (T-test: p = 1.46*10^− 3^; t = 3.83) When comparing the weekly diversity to those seen in week one, the New Orleans site averaged a Bray-Curtis dissimilarity of 0.592, while the North Shore averaged 0.288. Again, these differences were determined to be statistically significant. (T-test: p = 2.55*10^− 4^; t = 4.67)

### Total taxa turnover

**Figure 2:**
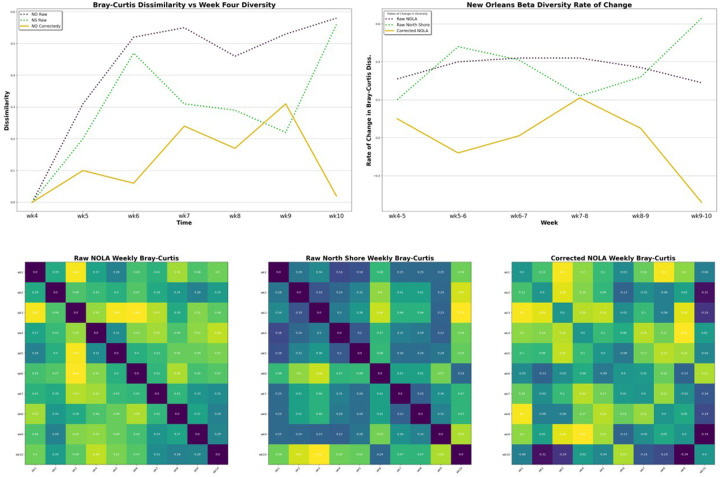
Change in Beta Diversity (top left) Bray-Curtis dissimilarity compared to week 4 diversity measures. (top right) Weekly rate of change in diversity using Bray-Curtis dissimilarity. (bottom row) heatmaps for Bray-Curtis dissimilarity for (left) uncorrected New Orleans, (center) uncorrected North Shore, and (right) corrected New Orleans.

Bray-Curtis dissimilarity was also measured for all taxa. (**see** Fig. 2) Weekly species turnover steadily increased over weeks 6-to-8 before gradually decreasing over weeks 8-to-10. Bray-Curtis dissimilarity between week four diversity and each subsequent week gradually increased and peaked at week nine, with a smaller peak at week seven. This steady rise in dissimilarity had a strong, significant, positive correlation with progression through Carnivale. (R = 0.895, p = 0.0160)

### Alpha Diversity

**Figure 3:**
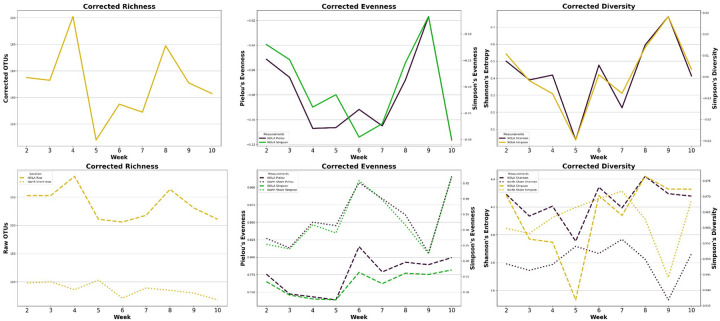
Change in Alpha Diversity (top row) change throughout Carnival for New Orleans’s alpha diversity metrics corrected for weather variation. (bottom row) uncorrected alpha diversity values for New Orleans and the North Shore. (left column) species richness i.e. OUT counts, (center column) Pielou and Simpson’s Evenness, and (right column) Shannon’s Entropy and Simpson’s Diversity index. Week one was omitted as the diversity measures of week one North Shore were substantially low compared to others, enough to be easily ruled an outlier.

Following week four, species richness slowly increased 65.7% until peaking at week eight – about ten days following Mardi Gras Day – and then began a gradual decline. (**see** Fig. 3, **Supp. Tables 3**) All metrics for evenness and overall diversity reached peak values at week nine – over two weeks after Mardi Gras Day. Pielous’s evenness increased 84.3% from its week four minimum, while Simpson evenness increased 77.4% from its week six minimum. Shannon entropy and Simpson diversity index increased 1,967.8% and 195.4% from their week five minimums, respectively. When we focused on weeks five through nine, we found that changes in all alpha diversity measures had strong, positive correlations with progression through Carnivale, while only Simpson diversity index reached statistical significance. (**see Supp. Table 4**)

### Pathogen tracker

**Figure 4:**
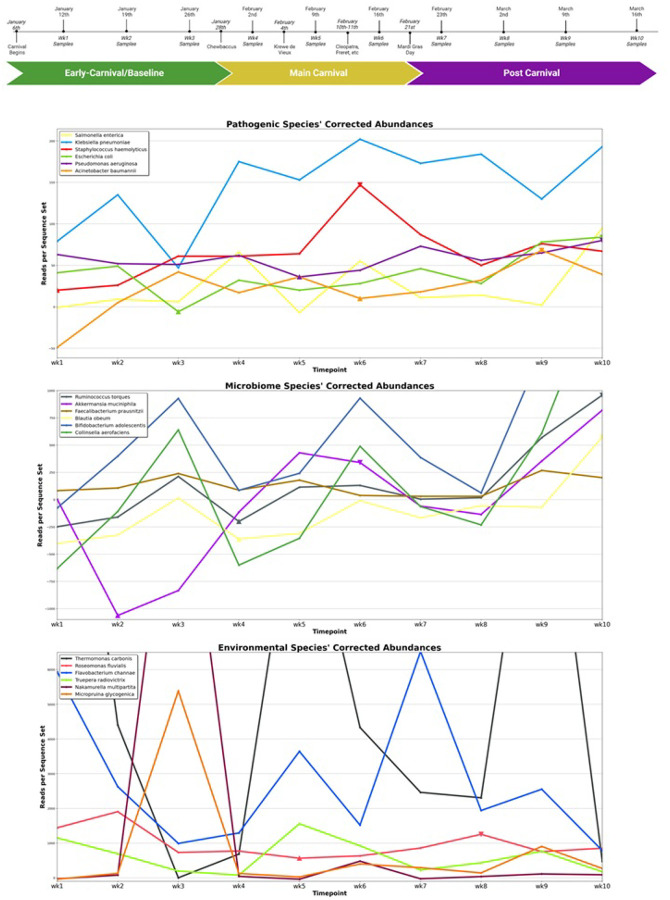
Changes in abundance of individual species over Carnivale. Abundance over Carnivale season of select (top) pathogens of interest, (center) dominant human gut microbiota and (bottom) environmental bacteria common in water and soil. Periods of the most significant increase in concentration that have a strong, positive correlation with the progression through Carnivale are indicated with upwards triangles at their start and downwards triangles at their conclusion.

We calculated the periods of peak significance between a species’ change in wastewater concentration and progression through Carnivale season using linear regression. The means of these values for our pathogen, microbiome, and environmental control groups varied significantly from one another. (one-way ANOVA: F-statistic = 7.23, p = 6.34*10^− 3^) (**see** Fig. 4, Table 1, **Supp. Table 5**) Our six pathogens and six microbiota were most alike on average. (t-test: t-statistic = 0.888, p-value = 0.395) However, the peak significance values for the environmental bacteria, our negative control, differed significantly from each. (Pathogens vs environmental: t-statistic=−2.42, p-value = 0.0361; Microbiota vs environmental: t-statistic = 4.60, p-value = 9.77*10^− 4^)

To isolate and remove even more variation from weather, we ran multiple regression analysis between each species’ wastewater variation using the temperature and precipitation as covariates. We also standardized the period of interest to weeks 4–10. **(see Table 2)** Here too, the microbiota group varied the most from the environmental control group, with the differences in the mean significances between their wastewater concentrations and Carnivale progression for both groups nearly being significant itself, while the difference between the pathogens and the environmental controls were not significant. (Microbiota: p = 0.061; Pathogens: 0.304) However, the difference in the t statistics for both experimental groups was significant as compared to the control group, (Microbiota: p = 0.034, Pathogens: p = 0.003) with the environmental group having a negative mean t statistic while the other two groups were strongly positive on average.

### CDC BEAM Monthly Clinical Isolate Data

We collected clinical isolate data from the CDC’s BEAM (Bacteria, Enteric, Ameba, and Mycotic) Dashboard to confirm whether rises in wastewater concentrations coincided with rises in symptomatic cases. (**see** Fig. 3) Two taxa were able to be compared tangentially: BEAM’s *Salmonella spp*. isolates to our wastewater *S. enterica*, which failed to increase significantly in wastewater concentration over the 2023 Carnivale season, and BEAM’s STEC to our wastewater *E. coli*, which showed the most significant increase of all the species concentrations that we tracked. All clinical isolate data underwent min-max normalization over a span of 0 to 1 so that states with vastly different population sizes could be accurately compared.

In 2023, Louisiana’s *Salmonella spp*. case isolates increased from 0 in February to 0.42 in March, a statistically significant increase when the surrounding states only rose an average of 0.11 over the same period. (Student’s t-test: p = 0.012) Louisiana saw an even more significant increase in STEC isolates, doubling from February to March. This represents a 0.5 increase when surrounding states decreased by 0.22. (Student’s t-test: p = 0.009)

To put these numbers into their proper context, we compared the 2023 isolate data to those of the 2021 season, in which all New Orleans’ Carnivale events were cancelled due to the COVID-19 pandemic. Louisiana’s *Salmonella spp*. isolates increased from a value of 0 in February to 0.7 in March. This *Salmonella spp*. increase is nearly as significant as it was in 2023, as the surrounding states only rose to an average of 0.16 in 2021. (Student’s t-test: p = 0.019) The 2021 STEC numbers, however, showed no increase in cases for Louisiana while the surrounding states also remained mostly static, decreasing by a value of less than 0.01 between February and March. (Student’s t-test: p = 0.996)

## Discussion

It is documented that ambient temperature and precipitation can influence prevalence of both bacterial concentrations and microbial resistance genes in wastewater and other surface waters.^43–45^ To control for variation in the New Orleans sewerage bacterial concentrations and community diversity due to influence from weather, we attempted an experimental design for the study of mass gatherings by taking matched samples from New Orleans and the North Shore and using the difference between the two values as the weather-corrected measure of New Orleans’ sewage bacteria.

The justification for the use of bacterial concentrations on the North Shore as a control group is grounded in both the North Shore and New Orleans sampling locations sharing nearly identical temperatures and rainfall – being only thirty-five miles apart – but experiencing vastly differing tourism rates. Travelling between the two cities involves an hour-long drive across the world’s longest contiguous bridge, the Lake Pontchartrain Causeway, which is also a toll road. Due to this and the relative scarcity of hotels on the North Shore, we reason that tourism to the North Shore over Mardi Gras would be negligible in comparison to that in The Big Easy. To confirm that the weather in the two locations was sufficiently similar, we pulled out historical weather data for each sampling location and compared them. (**see Supp.** Figure 1, **Supp.** Table 1)

Indeed, New Orleans and the North Shore have extremely similar temperature and weekly precipitation. The various bacterial species’ concentrations measured had different degrees of correlation with the local weather during the 2023 New Orleans’ Carnivale season. Generally, our selected human pathogens’ abundances had extremely weak correlations with average weekly rainfall, with the absolute value of Pearson correlation coefficient for the various species and rainfall averaging a Pearson correlation coefficient of 0.120. There was a slightly stronger correlation between temperature and bacterial abundance, with the mean absolute value of correlation being R = 0.332. Our matched design allowed for us to reduce bacterial correlation with ambient temperature by 26.1%, down to a final mean Pearson correlation of R = 0.245.

By comparing weekly species turnover we demonstrated that the rate of change in community composition for New Orleans was higher that that found in the North Shore, as we would expect for a community experiencing biotic invasion. (**see** Figs. 1&2) Furthermore, we found that the rate of change in the New Orleans’ community composition increased slowly over weeks 6-to-8 before peaking and gradually declined over weeks 8-to-10. This peak occurs a little over a week after Mardi Gras Day. Community diversity metrics strayed from baseline levels the most during weeks nine and seven. This seems to demonstrate that some species peaked in diversity during Mardi Gras, while others continued to slowly increase over the following 2-to-3 weeks.

All diversity measures tested increased from week 5-to-9, i.e. two weeks prior to Mardi Gras Day to over two weeks post. (**see** Fig. 3). This is expected if tourism is indeed importing new species to the New Orleans’ population, as new species means increased richness and even increased evenness initially if the imported bacteria exist in similar abundances in their own endemic populations. The time frame, weeks 5 through 9, appears to coincide with the peak of Carnivale festivities from weeks 4 to 7 when we factor in the apparent 2–3-week lag established elsewhere in this paper and others.^4,19,49^

Of most interest to public health surrounding mass gatherings, all our alpha and beta diversity metrics were returning to baseline levels by week ten, just over three weeks following Mardi Gras Day. This may indicate that public health interventions need only concentrate on the weeks during and directly after the mass gathering event. However, we would need to test further on post-Mardi Gras to make this claim definitively and ensure this was not a temporary aberration.

The pathogens selected are known human pathogens that have a documented predilection for acquiring ARGs, making them dually useful for both tracking of human disease and as sentinel species representing greater accumulation of ARGs. For example, if an abundance of E. coli – a common indicator species of ARG buildup – was to be found via 16S sequencing, it could signal that targeted PCR-based follow-up testing would be warranted to determine if there was a rise in particular pathogenic strains, e.g. ETEC, STEC, etc, or if particular antibiotics-resistance genes were present in those populations, e.g. AmpC, TetA, etc.^6^

We looked at two other populations of species typical of municipal wastewater. As a negative control, we looked at six environmental bacterial species, i.e. bacteria that do not typically colonize humans and whose abundance in municipal wastewater should be unaffected by human migration. We performed t-tests between each species and progression through Carnivale. When we averaged the t statistics, which indicates both the magnitude and the direction of the relationship, our environmental controls had an average t statistic of −0.534, indicating on average a weak, negative relationship between the species’ concentration and progression though the heart of Carnivale season.

Among the six selected environmental species, we only measured one period of significant, strongly correlated increase in wastewater concentration over Carnivale season: *Vibrio fluvialis*. (**see** Fig. 4, **Supp. Table 5**) Perhaps not coincidentally, *V. fluvalis* does have a documented ability to opportunistically infect the human gut, despite typically preferring coastal waterways.^46^

The second group we tracked were six common members of the human gut or urogenital tract microbiota, a category that makes up a major proportion of municipal waste bacteria.^47^ These species are certainly imported en mass during large-scale movements of humans. However, these species are not generally known to commonly induce pathogeneses which would increase their transmission from person to person i.e. conditions like UTIs or diarrhea that require an incubation time to manifest. Our microbiota had a mean t statistic of 2.07. Here we see a statistically significant difference between the mean t statistic of our environmental negative controls. (p = 0.003)

One species of human microbiota, *F. prausnitzii*, had a period of significant, strongly negative correlation between its wastewater concentration and the progression through Carnivale, the only microbiota species found to do so. Again, possibly not by coincidence, the prevalence of *F. prausnitzii* in the human gut has been found to decrease in abundance during periods of prolonged alcohol consumption.^48^

Turning then to our selected pathogens, we measured significant increases in abundances of nearly all human pathogens tracked over the course of late Carnival. The mean t statistic over the heart of Carnivale for this group was 1.97. Here again, this was statistically different from that of the environmental controls. (p = 0.034)

Such a gap between our two experimental groups and our environmental control suggests that the admixture of humans around mass gatherings has a significantly positive impact on the spread of common human colonizers and pathogens. This supposition is further supported by the observation that while a few of these pathogens peaked in abundance around weeks six or seven – the timepoints directly before and after of Mardi Gras Day – most peaked in abundance 2-to-3 weeks after, further mirroring the lag time seen in other studies of mass gatherings and contagious disease where the temporal dimension was analyzed. This is likely due to the incubation time between inoculation and shedding of bacteria.^19^ This contrasts our microbiome species, which typically peaked around week six. This may indicate that human microbiota are more directly impacted by importation into the community, as they typically occur at higher abundances in humans and do not need to rely on pathogenesis to spread and raise their wastewater profile.

**Figure 5:**
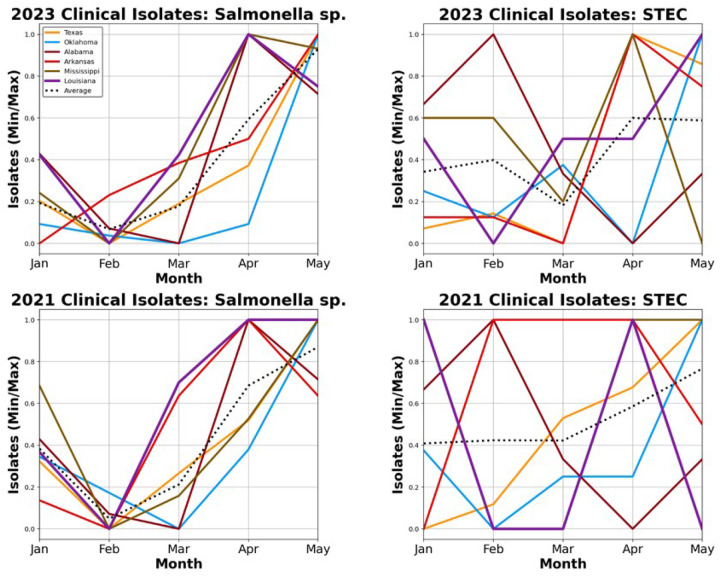
*Salmonella sp*. and STEC clinical isolates for selected states Min-max normalization of the number of clinical isolates per month collected in Louisiana and surrounding states for: (top left) all Salmonella species in 2023, (top right) STEC in 2023, (bottom left) all Salmonella species in 2021, and (bottom right) STEC in 2021. The average line is the average number of clinical isolates for all selected states surrounding Louisiana

Other studies that have explored the temporal correlation between mass gatherings, pathogen concentration in public wastewater and incidence of clinical isolates have found, on average, a 1–4 week lag between the mass gathering event and a rise in wastewater concentration and 2–3 week lag between the event and increasing clinical cases, depending on the length of incubation period for that particular species.^4,19,49^ Mardi Gras and the crescendo of Carnivale was not until the third week of February and most pathogen wastewater concentrations did not peak until early in the second week of March. (**see** Fig. 5) Therefore, we would expect to see a large spike in clinical isolates from March relative to February for any species which underwent significant wastewater spikes in the beginning of that month.

Compared to every state surrounding it, Louisiana had significant increases in clinical isolates of both *Salmonella spp*. (compared to wastewater: *S. enterica*) and STEC (compared to wastewater: *E. coli*). (**see** Fig. 4) However, *S. enterica’s* increases in wastewater concentration failed to significantly correlate to progression through Carnivale season, while *E. coli* showed the strongest wastewater correlations of all pathogens tracked. To shed light on this apparent contradiction, we looked at the same clinical isolate metrics from just two years prior, when the COVID-19 outbreak cancelled Carnivale festivities in New Orleans for the first time since the late 1970s.

Here we saw clinical isolates of *Salmonella spp*. increase in early Spring without Carnivale tourists just as significantly as they had when they were present, suggesting that the increases in clinical cases, and very well the moderate increases in wastewater, are likely due to a different phenomenon, such as seasonality. However, in the year without Mardi Gras, Louisiana’s STEC clinical cases were indistinguishable from those of the surrounding states, while in the 2023 Carnivale season Louisiana was significant in both the number of March STEC isolates and the increase in isolates from February to March when compared to all surrounding states. Being as the *E. coli* wastewater correlation was also robust, it is hard to argue that Mardi Gras tourism, the spike in wastewater *E. coli* concentrations, and that of STEC clinical isolates are not likely related.

## Supplementary Material

Supplementary Files

This is a list of supplementary files associated with this preprint. Click to download.

- supplementaryfiles.docx

## Figures and Tables

**Table 1:** Summary of correlation and significance of the relationship between individual species abundances and progression through Carnivale

Measure	Pathogens	Microbiota	Environmental
Peak Periods			
*Mean Significance value (p-value)*	0.0513	0.0323	0.107
*Significance Standard Deviation (s*_*p*_ *)*	0.0466	0.0239	0.0317
Significance of Group vs Environmental Mean: p-values	0.0361	9.77*10^−4^	0
*Mean Correlation value (R)*	0.528	0.529	0.282
*Correlation Standard Deviation (s*_*R*_ *)*	0.682	0.664	0.847
Significance of Group vs Environmental Mean: Pearson Rs	0.591	0.586	0
*# Periods of Sig., Strong Corr. w/Mardi Gras*	8	4	1
*# Taxa with Sig., Strong, Positive Corr. w/MG*	4	3	1
Weeks 4-to-9			
*Mean Significance*	0.370	0.235	0.580
*Mean T Test Statistic*	1.968	2.069	−0.534
*Significance of Group vs Environmental Mean: p-values*	0.304	0.061	0.0
*Significance of Group vs Environmental Mean: t statistics*	0.034	0.003	0.0

Table 1: (Peak Periods) Average peak Pearson correlations and linear regression significances of selected species’ wastewater concentration with progression through Carnivale. (Weeks 4-to-9) Average Pearson correlations and linear regression significances of each experimental group over weeks 4-to-9, the peak of the variation due to tourism.

## Data Availability

All raw sequencing reads datasets were deposited in GenBank SRA under the BioProject PRJNA1363154.
